# Unconventional site of pacing for failed balloon valvotomy of prosthetic tricuspid valve: a case report

**DOI:** 10.1186/s43044-024-00598-9

**Published:** 2025-01-06

**Authors:** Aditi Newaskar, Radha Nandipati, Somasekhar Ganta, Dharma Teja Dhulipalla, John Satish, Krishna Prasad Nevali

**Affiliations:** 1 Department of Cardiology, Sri Jayadeva Institute of Cardiovascular Sciences, Bengaluru, India; 2 Department of Cardiology, NRI Academy of Sciences, Guntur, India

**Keywords:** Epicardial pacing, Tricuspid valve replacement, Infective endocarditis, Coronary sinus pacing

## Abstract

**Background:**

Conduction disturbances are a frequent occurrence after tricuspid valve surgeries, and their management is challenging.

**Case presentation:**

We present a case of 16-year-old male patient who presented with episodes of presyncope. At the age of 7 years, he underwent tricuspid valve replacement surgery with a biological prosthesis for infective endocarditis sourced from a gluteal abscess. Post-operatively, he had complete atrioventricular block and underwent epicardial pacing. Epicardial lead impedance increased at follow-up and led to failure of pacing. At the time of presentation he had prosthetic tricuspid valve dysfunction with increased gradients. We did tricuspid valve balloon valvotomy but the gradients did not improve and patient developed tricuspid regurgitation. In view of difficulty in placing the lead through degenerated tricuspid valve, we have decided to place the lead in coronary sinus. He underwent successful lead placement in posterolateral tributary of coronary sinus with acceptable parameters. He had an uneventful follow-up at 1 year.

**Conclusion:**

TV surgeries create a unique problem for pacing. Coronary sinus pacing offers an effective alternative to conventional RV pacing in such cases.

**Supplementary Information:**

The online version of this article (10.1186/s43044-024-00598-9) contains supplementary material, which is available to authorized users.

## Background

Tricuspid valve (TV) infective endocarditis (IE) is uncommon compared to left-sided IE. Many times, these patients need valve replacement, and usually a biological prosthesis is inserted. TV surgeries are more prone to develop conduction disturbances owing to its proximity to the conduction system. Conventional right ventricular (RV) pacing in these circumstances is difficult, detrimental causing accelerated degeneration of the prosthetic valve and also increases the prosthetic valve stenosis and regurgitation [1]. Left ventricular pacing via coronary sinus offers an effective alternative in these circumstances. Bioprosthetic valves are prone to degeneration as age progresses and the risk is higher in younger ages. Balloon valvotomy is an alternative to delay redo tricuspid valve replacement (TVR) [2]. We present a 16 year old with TV prosthetic valve dysfunction requiring pacing for complete AV block and underwent CS lead implantation.

## Case presentation

**Fig. 1:**
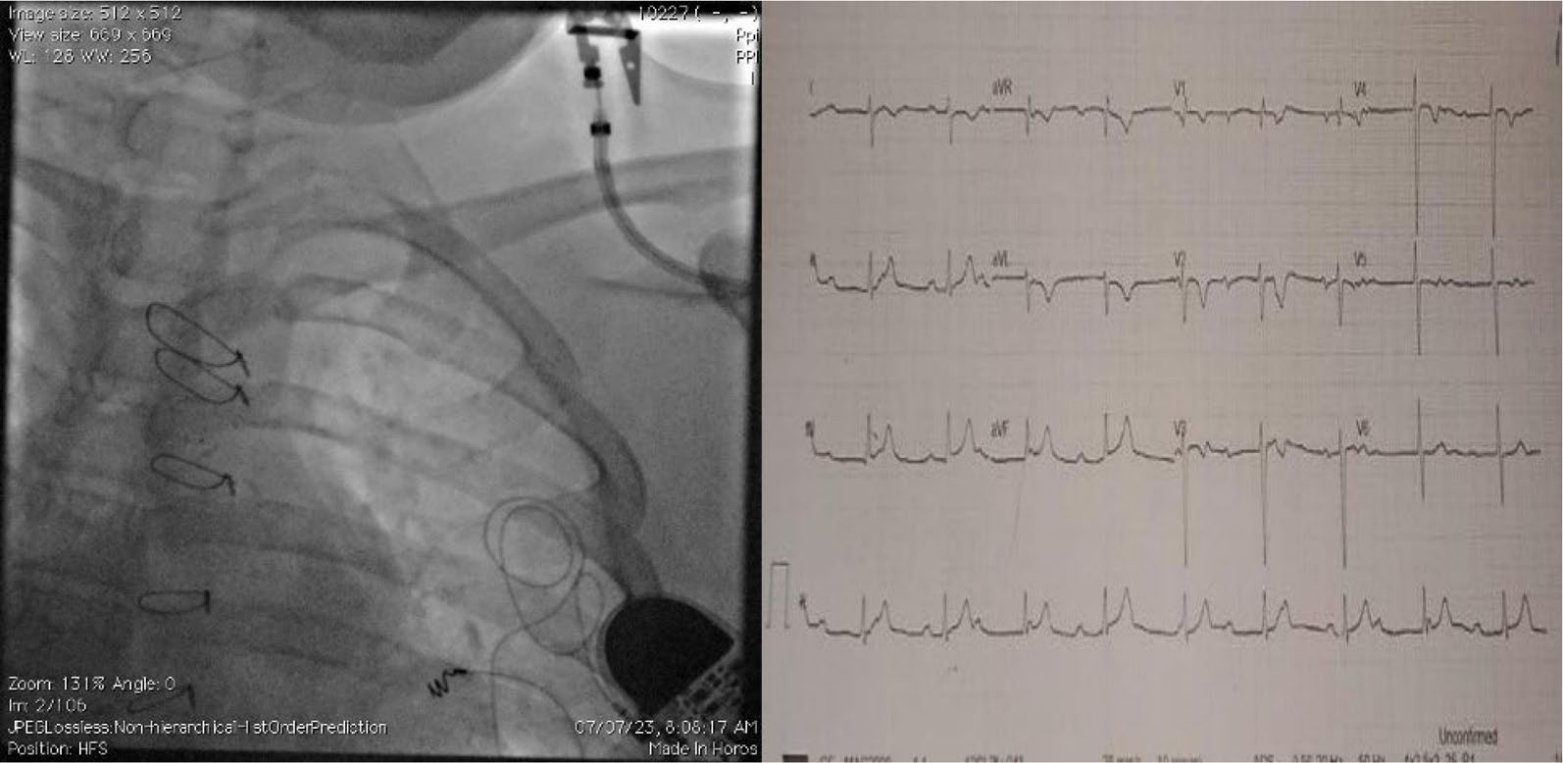
A fluoroscopy image showing unraveled spring of the epicardial lead. B Electrocardiogram showing complete atrioventricular dissociation with a rate of 100 per minute and V rate of 50 per minute)

This 16-year-old male has a complex cardiac history, starting with a left gluteal abscess at age 7, likely caused by trauma, which led to tricuspid valve vegetations and right ventricular (RV) dysfunction, possibly due to infective endocarditis. Despite 4 weeks of IV antibiotics, his RV dysfunction and tricuspid regurgitation (TR) persisted, necessitating tricuspid valve replacement (TVR) with a BIOCOR tissue valve. Postoperatively, he developed complete AV block, requiring implantation of a permanent pacemaker with an epicardial lead. Seven years later, the pacemaker’s pulse generator reached end of service, requiring a replacement, but the lead parameters were still normal. One year after this, he presented with syncopal episodes, and pacemaker interrogation revealed increased lead impedance and elevated pacing thresholds, with fluoroscopy showing an unraveled epicardial lead, indicating lead malfunction (as shown in Fig. 1A). The diagnosis is epicardial lead failure, likely due to mechanical issues, which is preventing adequate pacing. The next steps involve replacing the malfunctioning lead, reprogramming the pacemaker, reassessing cardiac function, and continuing long-term follow-up to monitor for any future complications, including infective endocarditis or further pacemaker issues.

**Fig. 2:**
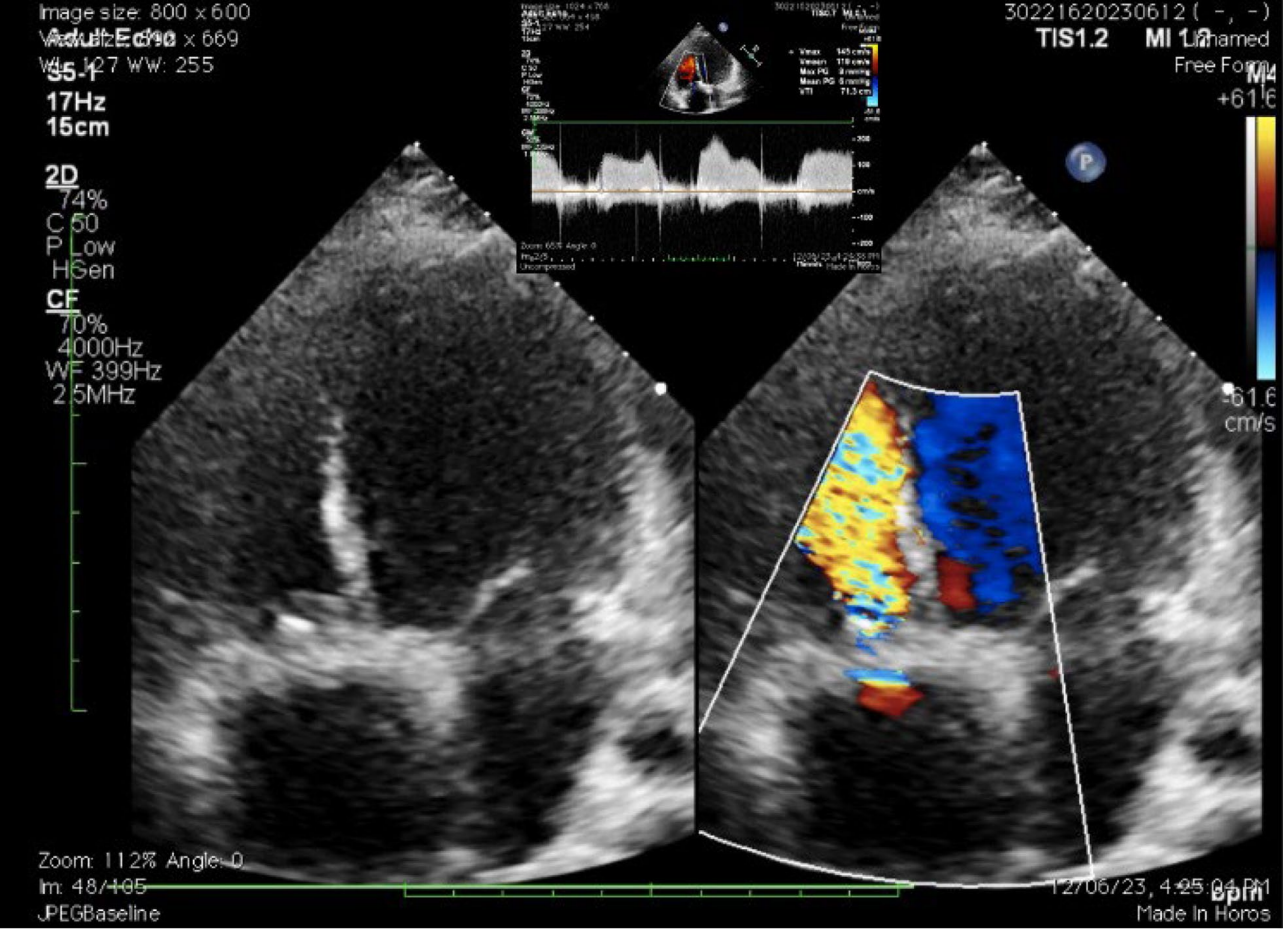
Echocardiogram showing apical 4 chamber view with color Doppler showing turbulent flow across tricuspid valve (off-set image showing continuous wave Doppler tracings with peak gradient of 8 mm Hg and mean gradient of 4 mm Hg)

Electrocardiogram showed complete AV dissociation (as shown in Fig. 1B). Echocardiogram at this stage showed degenerated prosthetic valve with increased gradients with no regurgitation (mean gradient across TV = 6 mm Hg) (as shown in Fig. 2 and Video 1). However, patient has no right-sided heart failure symptoms. So it was decided for new lead placement.

We have discussed the following options with the caregivers.

1. Placement of new epicardial lead with or without redo TVR.
2. Placement of endocardial RV lead across the degenerated TV.
3. Balloon dilatation of TV followed by endocardial lead placement across the TV.
4. Placement of CS lead for LV pacing.
5. Use of leadless pacing system.

As per caregivers choice, we went ahead with percutaneous balloon dilatation of TV. (Even though he was asymptomatic, we thought it will delay the repeat valve replacement and allows us to place the conventional lead across the TV.) CS lead was not considered initially due to financial reasons and stability issues in a pacing-dependent patient. Leadless device was not considered due to financial constraints.

### Procedure details

**Fig. 3:**
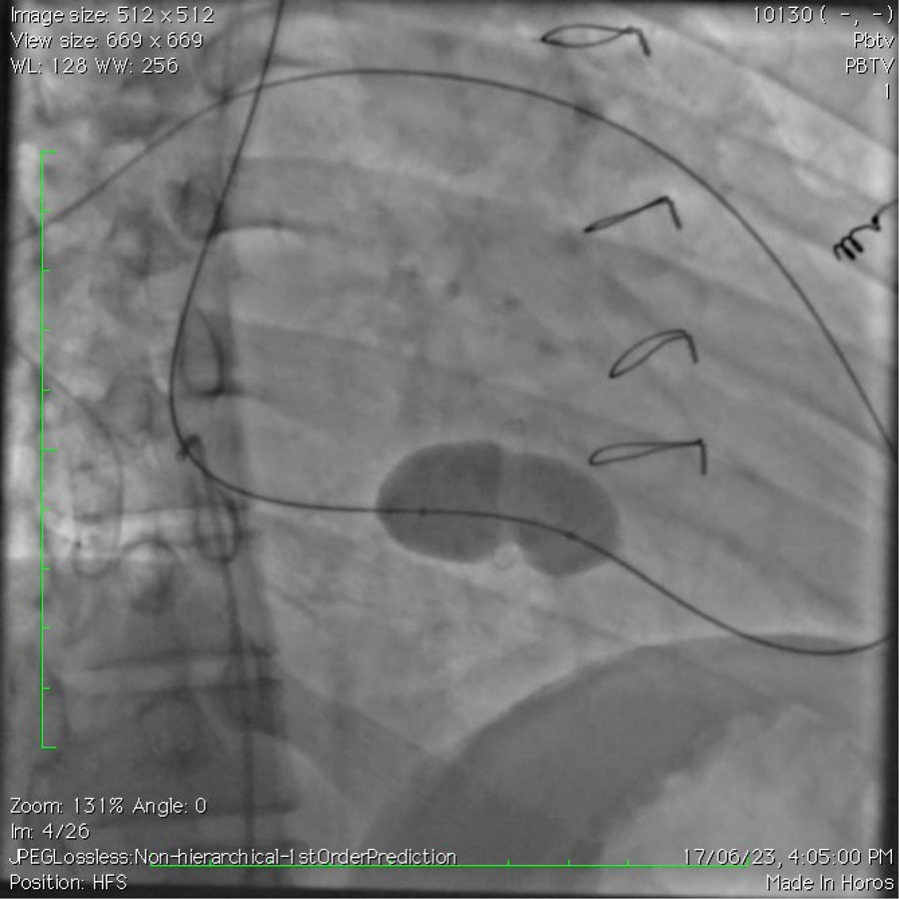
Fluoroscopy image showing Tyshak balloon being inflated across tricuspid valve

Jugular access was taken and a multipurpose (MP) catheter was used to cross the TV, and pulmonary valve. Then, an Amplatz super stiff wire was parked distally in the pulmonary artery branches. Then, a 20 × 40 mm Tyshak Balloon (NuMED™) was inflated across the TV (as shown in Fig. 3) (Video 2). However, gradient decreased only by 1 mm Hg and patient developed TR (Video 3). Hence, further dilatations were not done. Possibly the degenerated lead was calcified and did not yield much, but in view of development of TR, further dilatations were not attempted.

**Fig. 4:**
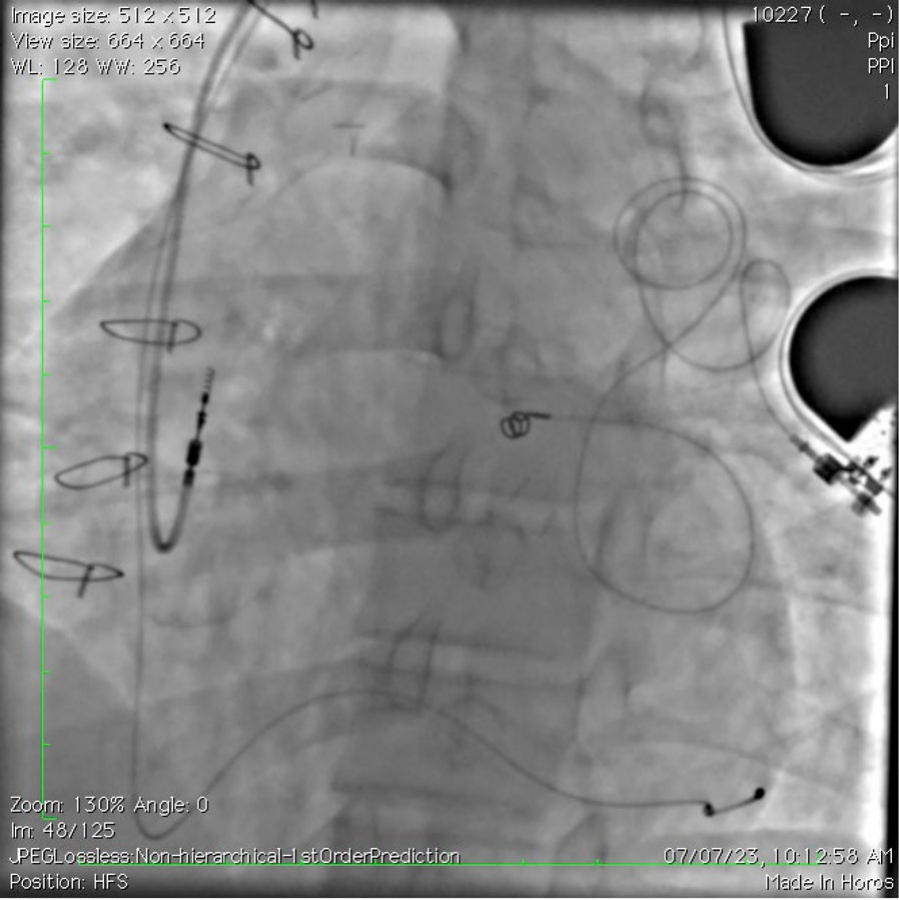
Fluoroscopy image showing dual-chamber device with right atrial lead in right atrial appendage and ventricular lead in one of the coronary sinus tributaries

As the caregivers did not consent for epicardial lead placement, we have decided to do LV pacing by implanting a lead in CS tributaries. A decapolar catheter was used to cannulate the CS, and a slittable MP sheath was placed in the CS. Then, a bipolar LV lead (Medtronic, USA) was placed in the posterolateral vein with good parameters. Then, an RA lead was positioned with good parameters. A dual-chamber generator was connected (as shown in Fig. 4 and Video 4). At 1-year follow-up, patient had good parameters and is doing well.

## Discussion

Right-sided IE accounts for approximately 5–10 percent of all cases of IE. TVR is the treatment of choice when there is poor response to intravenous antibiotics [3]. Our patient had TV IE at the age of 8 years, and it was unresponsive to antibiotics and underwent TVR by a bioprosthetic valve.

TV surgeries are usually associated with high percentage of atrioventricular blocks (AVB) due to anatomical proximity of the atrioventricular node and the conduction tissue to the tricuspid annulus. The conduction tissue and the anteroseptal commissure of the TV are closely located, resulting in a high risk of His bundle trauma during TV surgery. Injury to the node or even edema of the surrounding tissue could cause temporary or permanent AVB [4]. Our patient developed AV block postoperatively and underwent permanent epicardial pacing as it was appropriate for his age.

Inaccessible or difficult entry into RV is seen with prosthetic valves, univentricular hearts, and Fontan circulation and poses a challenge for permanent pacing. Sometimes, it is not the difficulty in RV entry but the presence of a lead across the TV and the lead-induced TR may hasten the process of bioprosthetic or native valve degeneration.

In such cases, epicardial pacemaker implantation is a better approach especially when the child is young. However, epicardial pacemaker lead implantation is usually associated with development of high thresholds at follow-up and has its own challenges [5]. In our patient at follow-up, he had increased threshold, and in fluoroscopy, epicardial lead fracture or unraveling was observed. So we have planned an endocardial lead placement.

Bioprosthetic valve degeneration is not uncommon after 8 years. Balloon valvuloplasty may be partially effective by splitting the fused commissure. However, it may not be effective on a stiffened valve base caused by heavy pannus or valve calcification. Redo TVR is usually necessary in the majority of the cases for definite treatment. In our patient after 8 years of TVR, there were increased gradients, and we reckoned balloon valvuloplasty might temporarily benefit the patient and delay redo TVR and can help in placing the pacing lead across the TV.

As balloon valvotomy did not have much effect on the gradients and had increase in the TR, we thought putting a lead across the degenerated bioprosthetic TV will accelerate the valve degeneration.

In this scenario, pacing the LV through the CS tributaries was considered as the best option to avoid surgical reintervention, or an endocardial lead through the TV bioprosthesis. There were few cases described where lead was placed in CS and use of leadless pacemaker in patients with TV prosthesis [6].

This case is unique; in that, we attempted balloon valvotomy for TV bioprosthesis degeneration albeit with limited success and pacing the LV through CS tributaries avoiding the valve.

## Conclusion

TV surgeries create a unique problem for pacing. Coronary sinus pacing offers an effective alternative to conventional RV pacing in such cases.

## Supplementary Information

Additional file 1

Additional file 2

Additional file 3

Additional file 4

## Data Availability

The dataset is available with the authors and can be provided on reasonable request.

## Abbreviations

AVB: Atrioventricular Block
CS: Coronary sinus
IE: Infective endocarditis
LV: Left Ventricle
PGR: Pulse Generator Replacement
RV: Right Ventricle
TV: Tricuspid valve
TVR: Tricuspid Valve Replacement
TR: Tricuspid Regurgitation
